# Supported TiO_2_-ZnWO_4_ Photocatalytic Nanofibrous Membranes for Flow-Through and Fixed-Bed Reactors

**DOI:** 10.1021/acsomega.3c03527

**Published:** 2023-08-09

**Authors:** Nakarin Subjalearndee, Pasinee Panith, Tanaporn Narkbuakaew, Pech Thongkam, Varol Intasanta

**Affiliations:** National Nanotechnology Center, National Science and Technology Development Agency, 111 Phahonyothin Road, Klong Nueng, Klong Luang, Pathumthani 12120, Thailand

## Abstract

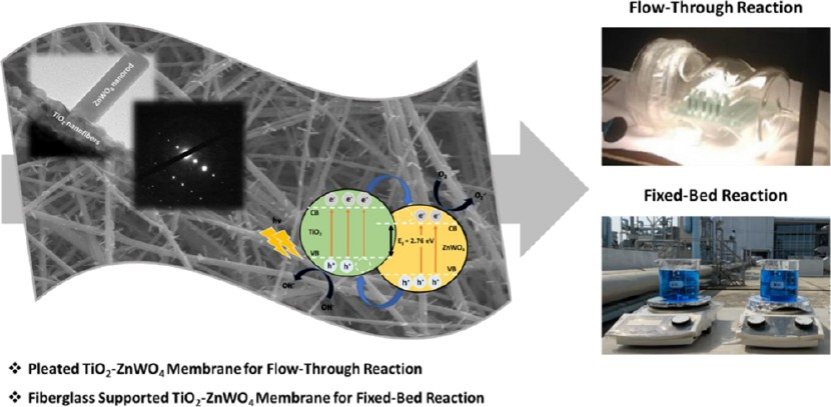

We developed utilization models of supported electrospun TiO_2_-ZnWO_4_ photocatalytic nanofibrous membranes for air and water purifications using a noncomplex system with facile adaptation for large-scale processes. For this uniquely designed and multimode catalyst, ZnWO_4_ is selected for a visible light activity, while TiO_2_ is incorporated to enhance physical stability. Morphological structures of the TiO_2_-ZnWO_4_ membrane are characterized by scanning electron microscopy and scanning electron microscopy–energy-dispersive X-ray spectroscopy. The distinguished growth of ZnWO_4_ nanorods at the surface of the TiO_2_-ZnWO_4_ membrane is revealed by transmission electron microscopy (TEM). The relaxation process and charge transfer mechanism are proposed following the examination of interface and band gap (2.76 eV) between TiO_2_ and ZnWO_4_ particles via HR-TEM and UV–vis spectrophotometry. For the gas-phase reaction, a transparent photocatalytic converter is designed to support the pleated TiO_2_-ZnWO_4_ membrane for toluene decomposition under visible light. To obtain a crack-free and homogeneous fiber structure of the pleated TiO_2_-ZnWO_4_ membrane, 1 h of nanofibrous membrane fabrication via a Nanospider machine is required. On the other hand, a fiberglass-supported TiO_2_-ZnWO_4_ membrane is fabricated as a fixed-bed photocatalyst membrane for methylene blue decomposition under natural sunlight. It is observed that using the calcination temperature at 800 °C results in the formation of metal complexes between fiber glass and the TiO_2_-ZnWO_4_ membrane. The TiO_2_-ZnWO_4_ membrane successfully decomposes toluene vapor up to 40% under a continuous-flow circumstance in a borosilicate photocatalytic converter and 70% for methylene blue in solution within 3 h. Finally, the mechanically robust and supported TiO_2_-ZnWO_4_ nanofibrous membranes are proven for an alternate potential in environmental remediation.

## Introduction

Water and air pollutions contribute to the most threatening environmental issues worldwide, leaving opportunity costs to the global society as a whole. Automobile releases of volatile organic compounds are ones of the most serious threats to air quality, climate change, and the future of our planet.^1,2^ Among various types of air pollutants produced from automobile exhaust, volatile organic compounds (VOCs) such as toluene have been mentioned as ones of the most toxic substances. With a severe or long-term exposure, VOCs can harm the environment and human health through respiratory and nervous systems.^3,4^ On the other hand, the excessive release of organic pollutants, such as methylene blue (MB) and rhodamine B (RhB) from dye industries, into the water resources remains prevalent.^5,6^ Therefore, effective measures to cure the environment are of scientific and technological interest.

Material solution is one of the most favorable answers to improve air and water quality. Considering promising materials for environmental application, photocatalysts with the well-known advantageous properties, e.g., safety to the ecological system and human health, are primarily mentioned. For instance, titanium dioxide (TiO_2_) has been studied and widely used as a photocatalyst in self-cleaning,^7^ degradation of organic pollutants,^8^ hydrogen evolution,^9,10^ energy conversion,^11^ and electrochromic device applications^12^ owing to their relatively high photocatalytic activity, chemical stability, and nonhazardous nature.^13−15^ The photocatalytic mechanism of TiO_2_ involves generation of electrons and positive holes in the conduction and valence bands under light irradiation. The generated holes can react with organic molecules or form hydroxyl radicals, while the electrons can reduce organic compounds.^16^ Another specific example of semi-conductor photocatalyst is zinc tungsten oxide (ZnWO_4_), which has been vastly utilized for organic pollutant removals.^17^ Generally, the photocatalytic mechanism of ZnWO_4_ concerns three steps under light irradiation: (i) generation of charge carrier pairs, (ii) migration of generated charge carriers on the surface of the photocatalyst, and (iii) redox reaction of oxidative and superoxide radicals.^18^ To enhance photocatalytic performance of conventional TiO_2_, heterojunction formation between TiO_2_ and metal oxides has been explored such as MoS_2_/TiO_2_^19^ and TiO_2_/ZnWO_4_.^20^ Specifically, the photocatalytic performance of TiO_2_/ZnWO_4_ significantly depends on the molar ratio between TiO_2_ and ZnWO_4_. Extensive difference in the molar ratio results in poor photocatalytic performance due to large defects in crystal grains.^20^ However, thermodynamically driven self-agglomeration could often be a problem due to their inherent high surface energy. Although the efficiency of nanostructured photocatalysts has been accepted, the utilization of these photocatalysts in the powder form still undergoes recovery problems as their small sizes make the separation impractical and costly. Therefore, the supporting substrate of photocatalysts becomes the significant part expected to solve their recoverability problems and utilization.

Among various approaches for supporting photocatalysts, using porous and flexible substrates such as paper-like substrates to sandwich the catalysts in flow-through or fixed-bed reactors has proven to be effective solutions.^21,22^ Significant advantages of both flow-through and fixed-bed reactors over the other techniques are noncomplex systems and facile adaptation for commercial process.^23^ In 2010, Koga et al. showed that Pt/Al_2_O_3_ catalyst powder could be successfully incorporated into a microstructured ceramic paper substrate. The unique hybrid structures could effectively mitigate NO_*x*_ in a flow-through reactor.^24^ In the following year, the team reported that immobilization of Pt and Cu nanoparticles on a microstructured paper-like matrix could be done via in situ synthesis of the metal nanoparticles on ZnO whiskers embedded in a ceramic paper matrix. The paper-like microstructure could promote effective transfer of heat and reactants to the highly efficient Pt nanocatalyst.^25^ Apart from the ceramic paper substrates, cellulose-based substrates have been recently used as a support for photocatalysts. In 2021, Sboui et al. prepared a hybrid cellulose paper-AgBr-TiO_2_ photocatalytic membrane via a direct adsorption procedure. The composite catalyst demonstrated effective photocatalytic performance in gas-phase ethanol degradation under stimulated sunlight irradiation.^26^ Recently, the RuO_2_/TiO_2_ nanocomposite on cotton fabrics had been developed as a photocatalytic membrane by coating hydrothermal treatment techniques. The photocatalytic membrane showed remarkable photocatalytic activity against o-toluidine and high mechanical durability.^27^ While the paper-like microstructures made possible extensive flexural deformation enabling the hybrid materials to play significant roles in various applications, the most important finding from these studies was that such special morphology led to effective transfer of heat and reactant. Even though the paper substrate might not stand practically high-temperature treatment, these studies have shed light into the key parameters which strongly influenced the catalytic conversion efficiency of fibrous structures.

In the present contribution, we proposed two comparable approaches to demonstrate utilization of a supported TiO_2_-ZnWO_4_ photocatalytic membrane for both air and water purifications under visible and natural sunlight. ZnWO_4_ was selected as a visible light active photocatalyst, while TiO_2_ was incorporated into the nanofibrous structures to enhance physical stability and photocatalytic efficiencies. Specifically, transparent photocatalytic reactor (TPR) was designed from borosilicate glass as a demonstrative photocatalytic air-phase converter applicable for supporting the TiO_2_-ZnWO_4_ nanofibrous membrane. For air purification application, the as-spun nanofibrous membrane was pleated along a track of glass and fiberglass support prior to high-temperature treatment inside a furnace to obtain an as-designed and mechanically stable TiO_2_-ZnWO_4_ nanofibrous membrane before evaluating the photocatalytic performance with a model toxin, toluene, under visible light irradiation. For the water-phase reaction, the fiberglass-supported nanofibrous membrane was calcined inside a furnace at the designated temperature. After the calcination process, we observed an unusual complex exchange between fiberglass and the nanofibrous membrane, forming mechanical support for the nanocatalyst. The photocatalytic performance of the fiberglass-supported TiO_2_-ZnWO_4_ nanofibrous membrane was then evaluated with MB solution under natural sunlight irradiation.

## Results and Discussion

### Fabrication of a TiO_2_-ZnWO_4_ Nanofibrous Membrane in a Transparent Photocatalytic Converter Reactor

The ZnWO_4_ nanofibrous membrane was initially fabricated via Nanospider machine in comparison with the TiO_2_-ZnWO_4_ nanofibrous membrane. Physical and chemical structures of the ZnWO_4_ membrane are shown in Figure S1 (Supporting Information). The as-spun nanofibers presented beads-free nanofibers with a diameter of ca. 152 nm (Supporting Information, Figure S1a,b). After calcination at 600 °C, the nanofibers presented fracture and high fragility (Supporting Information, Figure S1c,d). In comparison with TiO_2_-ZnWO_4_ nanofibers after calcination at 600 °C,^28^ differences in morphological structures were distinctly observed. Adding Ti component into the AMT and ZAH electrospinning solution before electrospinning resulted in transformation of spherical ZnWO_4_ nanoparticles into rod-shaped ZnWO_4_ nanoparticles along the TiO_2_ nanofibers. In addition to morphological structure alteration, TiO_2_ also enhanced flexibility of the TiO_2_-ZnWO_4_ nanofibers. Therefore, incorporating TiO_2_ into the TiO_2_-ZnWO_4_ nanofibers is mandatory to direct the growth of rod-shaped ZnWO_4_ particles and enhance overall flexibility of the TiO_2_-ZnWO_4_ nanofibers.

After fabricating the nanofibrous membrane from the solution containing AMT, ZAH, and TIP via Nanospider machine, the trial pleating experiment was initially performed under confinement from fiber glasses and glass slides. In the first experimental protocol, the membrane was cut into a 14 cm × 40 cm rectangular shape (Supporting Information, Figure S2a). The membrane was then pleated into an eight-compartment partition (Supporting Information, Figure S2b) before transferring inside a furnace for calcination at the designated condition. After calcination, the calcined membrane experienced drastic shrinkage and crumbling, and trapped strongly in between the fiber glasses, so the nanomembrane could not be removed from the support (Supporting Information, Figure S2c,d). It could be seen from this experiment that shrinkage could be a dominant phenomenon experienced by the nanomembrane, which could also strongly affect its own spatial arrangement. This observation agreed with some previous work involving thermal treatment of organic–inorganic hybrid materials or membranes into purely inorganic structure.^29,30^ It was found that while the organic components were degrading away, the developing inorganic components experienced extreme contraction and often were led to mechanical failure and dimensional fractures. Therefore, the heat-generated mechanical stress was considered, while the new reactor design tried to minimize that detrimental effect.

In the next experiment, the nanofibrous membrane was cut into the same shape and glass slides were introduced as partitioning support in the configuration and to help control the membrane shape during the calcination process (Supporting Information, Figure S3a,b). After calcination, the shrinkage of the calcined membrane moved the adjacent partitioning glass slides upward along with direction of shrinkage (Supporting Information, Figure S3c,d). Interestingly, the calcined membrane was able to withstand the weight of glass slides and no fragments were observed after the drastic dimensional change. Subsequently, the calcined membrane was characterized by scanning electron microscopy (SEM) to reveal the well-defined nanofibers and the unique characteristics of the nanorods at the surface (Supporting Information, Figure S3e). From the results, the pleated as-spun could be stabilized in a sandwich of fiberglass and partitioned by glass slides as rigid structural support. It could be said that the structural design successfully complied with the induced contraction to minimize the stress and avoid the unwanted mechanical failure.

**Figure 1:**
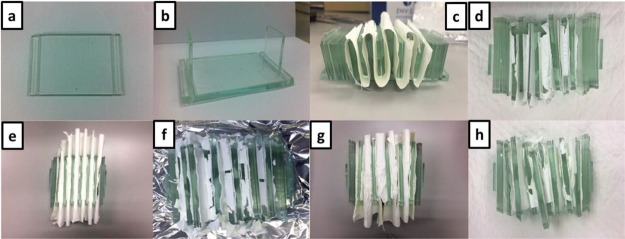
Pictures of supporting components for nanofibrous membrane pleating and pleating procedures. Pictures of (a) the plate part, (b) the plate part with supporting slides, experimental protocol 1 (c) before and (d) after calcination, experimental protocol 2 (e) before and (f) after calcination, and experimental protocol 3 (g) before and (h) after calcination.

We observed that the pleated and thermally treated nanofibrous membrane remained intact due to the structural support provided by such a rigid structure as glass slides. In this experimental trial, we further investigated the effect of the spacing provided by the glass slides. This aspect was expected to be important because the spacing influenced how evenly and thoroughly the membrane could interact with the model gas within the chamber. Experimentally, the sizes of spacing were varied by the number of the employed glass slides between 1 and 4, whereas the slides could be located above or below the pleating nanomembrane (Figure 1a,b). In addition, we also investigated the effect of the nanofibrous membranes’ thickness by varying the electrospinning time from 30 to 90 min (Table 1). First, the thinnest membrane (spinning time of 30 min, ca. 175 μm) was pleated using four glass slides on both upper and lower sides for membrane supporting (Figure 1c) in the experiment 1. Unfortunately, after the subsequent heat treatment, the thin nanomembrane fractured (Figure 1d). It was hypothesized that the all-inorganic nanofibrous membrane could not withstand the accumulating weight of glass slides on the upper side. In the following experiment (experiment 2), we employed a thicker nanomembrane thickness (spinning time of 60 min, ca. 201 μm) and the same number of glass slides on both sides (Figure 1e). From this arrangement, fractures were also observed (Figure 1f), but with a fewer independent fragments than the previous trial. Apparently, the better physical stability could well be ascribed to the larger thickness of the nanomembrane. In experiment 3, the thickness of the nanomembrane was increased (spinning time of 90 min, ca. 473 μm) in the presence of the sample supporting arrangement (Figure 1g). Incidentally, the respective calcined nanomembrane showed even more fractured structure than those from the previous two experiments (Figure 1h). It was hypothesized that the increased thickness translated into steric hindrance for the as-spun nanomembrane and unbearable stress projected onto the all-inorganic nanomembrane during calcination. It could be summarized that the four-glass-slide partitioning arrangement might not be suitable possibly due to the gravitational weight experienced by the nanofibrous membrane and that the thickness of the membrane could induce even more instability within the confinement.

**Table 1:** Summary of all Variations in Trial Pleating Experiments

experiment	nanomembrane spinning time	average thickness (gm)	approximate membrane size	types and number of supporting slides
1	30 min	175	10.5 × 34.5 cm	Upper side: 4 FGs and 4 GSs
				Lower side: 4 FGs and 4 GSs
2	60 min	201	6 × 42 cm	Upper side: 4 FGs and 4 GSs
				Lower side: 4 FGs and 4 GSs
3	90 min	473	10 × 34 cm	Upper side: 4 FGs and 4 GSs
				Lower side: 4 FGs and 4 GSs
4	30 min	173	11 × 36 cm	Upper side: 1 FG and 1 GS
				Lower side: 4 FGs and 4 GSs
5	60 min	307	10.5 × 36.5 cm	Upper side: 1 FG and 1 GS
				Lower side: 4 FGs and 4 GSs
6	90 min	405	9.5 × 34.5 cm	Upper side: 1 FG and 1 GS
				Lower side: 4 FGs and 4 GSs

**Figure 2:**
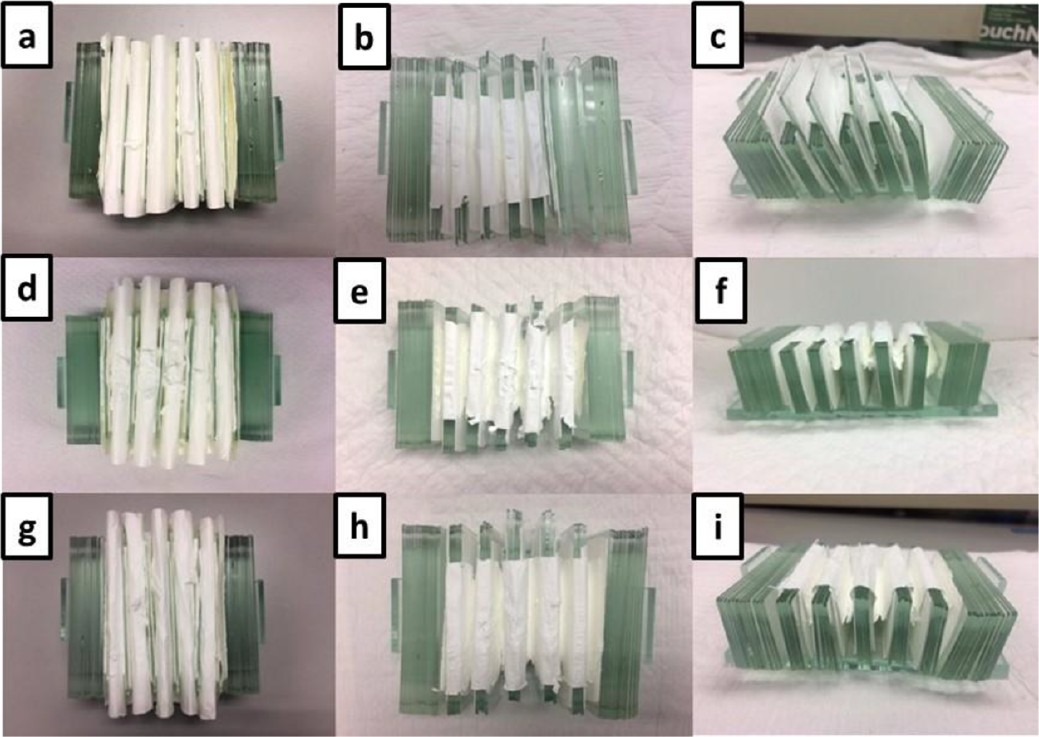
Pictures of pleated nanofibrous membranes on supporting components before and after calcination. Pictures of experimental protocol 4 (a) before and after calcination from (b) top (c) and side views. Pictures of experimental protocol 5 (d) before and after calcination from I top (f) and side views. Pictures of experimental protocol 6 (g) before and after calcination from (h) top (i) and side views.

In the following experimental sets (experiment 4–6), we reduced the weight on the inorganic membrane by reducing the number of glass slides on the upper side from four to one slide, while controlling other parameters such as membrane thickness and the number of glass slides on the lower side (Figure 2a). Interestingly, the membrane after calcination showed stable physical structure without any fragmentation observed from both top and side views in experiment 4 (Figure 2b,c). We then increased the thickness of the nanomembrane (spinning time of 60 min, ca. 307 μm) as illustrated in Figure 2d. After calcination, the inorganic nanomembrane with thickness of ca. 307 μm was moderately fractured right at the corner of the pleat in experiment 5 (Figure 2e). However, this membrane was less sensitive to handling as no displacement was discerned (Figure 2f). In the last trial, the nanomembrane with the spinning time of 90 min (ca. 405 μm) was studied (experiment 6). However, its pleating process was difficult due to the thickness of the membrane (Figure 2g). Nevertheless, the nanomembrane presented a high level of stability (Figure 2h,i) upon calcination. From these results, experimental protocols 4–6 were explored further.

**Figure 3:**
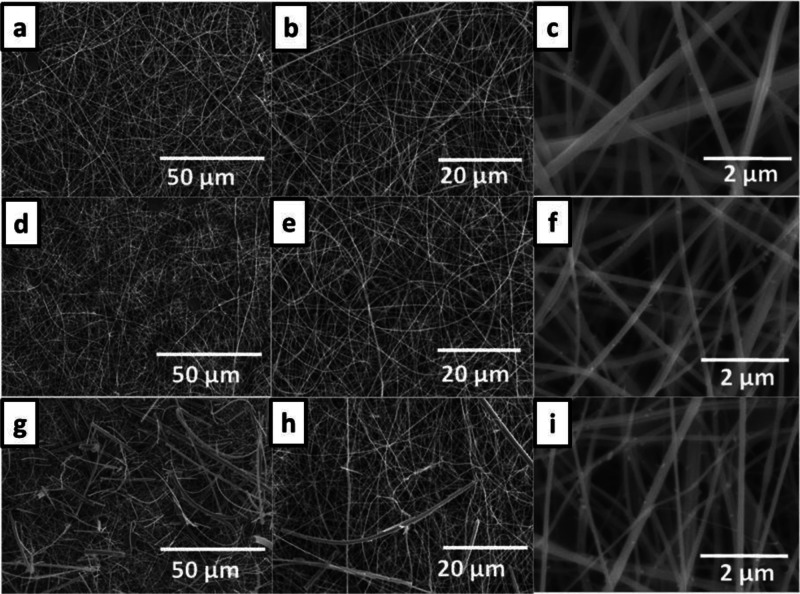
Morphological characterization of the TiO_2_-ZnWO_4_ nanofibrous membrane before and after calcination under different confinement conditions. SEM images of TiO_2_-ZnWO_4_ nanofibers after calcination in experimental protocol (a–c) 4, (d–f) 5, and (g–i) 6.

**Scheme 1:**
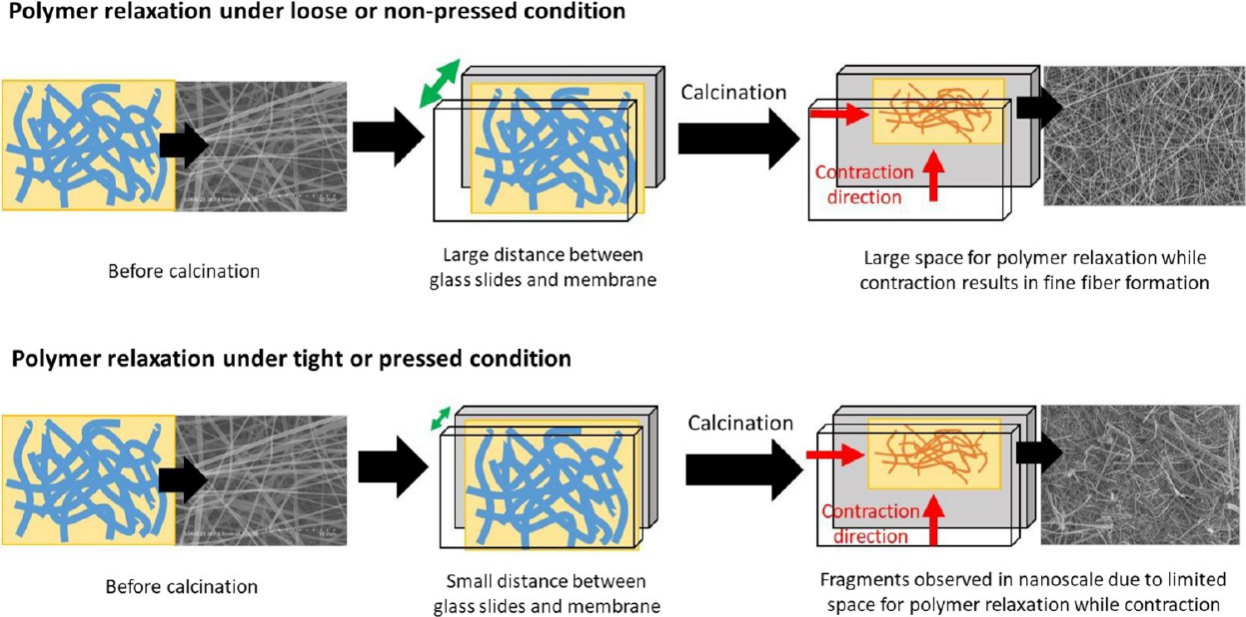
Mechanical Failure of the TiO_2_-ZnWO_4_ Nanofibrous Membrane During Formation

Apart from the macroscopic examination of the samples, the SEM unveiled the physical characteristics of constituting nanofibers. The specific purpose was to observe the overall and local area of nanofibers, so SEM images were taken from 50- to 2-μm size projection. The sample from experiment 4 showed crack-free membrane and homogeneous and bead-free fiber structure (Figure 3a,b). At the higher resolution, the nanofibers presented a unique characteristic of nanorods along the surface (Figure 3c). The sample from experiment 5 with thicker membrane appeared similar to the nanorods (Figure 3d–f). On the other hand, the sample from experiment 6 showed discernable fragmented nanomembrane (Figure 3g,h), which could be since the thick membrane led to strong stress and strain build up during the high-temperature treatment as hypothesized in Scheme 1. Nevertheless, on the nanoscopic view, the nanofibers remained intact like those from the previous protocols (Figure 3i). From these results, it could be seen that the sample from experiment 5 was the most stable which would be selected for the following study.

**Figure 4:**
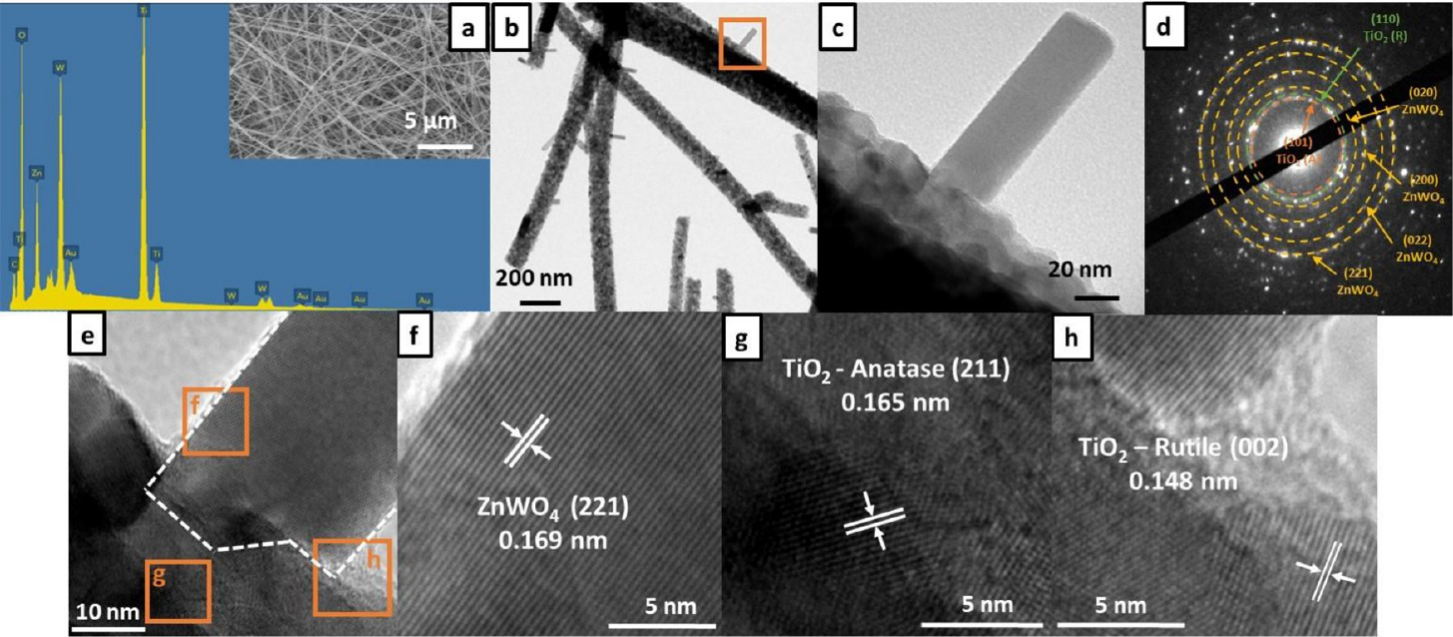
Morphological and chemical structures of confined TiO_2_-ZnWO_4_ nanofibrous membrane. (a) SEM–EDX spectrum, (b and c) HR-TEM images, (d) SAED measurement, and (e–h) selected HR-TEM images of TiO_2_-ZnWO_4_ nanofibers.

Morphological and chemical structures of the TiO_2_-ZnWO_4_ nanofibrous membrane from the experiment 5 were further characterized by scanning electron microscopy–energy-dispersive X-ray spectroscopy (SEM–EDX) and HR-TEM (Figure 4). The SEM–EDX spectrum showed that Zn, W, Ti, and O were main chemical compositions of the nanofibers (Figure 4a and inset). TEM image revealed that the TiO_2_-ZnWO_4_ nanofibers composed of two major phases of nanofibers and rod-like particles (Figure 4b,c). Anatase and rutile TiO_2_ and ZnWO_4_ were observed after conducting selected area electron diffraction (SAED) (Figure 4d). After analyzing lattice planes of the TiO_2_-ZnWO_4_ nanofibers (Figure 4e), it was observed that anatase and rutile TiO_2_ were main components in the nanofibers, while rod-like particles were ZnWO_4_ (Figure 4f–h) (JCPDS card no. 03-065-5714, 01-071-6411, and 01-089-0447). The interface between ZnWO_4_ nanorod and TiO_2_ nanofibers was indicated in Figure 4e (white line). Interaction between TiO_2_ and ZnWO_4_ was confirmed by X-ray diffraction (XRD). It could be observed that crystallinity of TiO_2_ such as the (110) crystal plane was suppressed by introduction of ZnWO_4_ crystal structures (Supporting Information, Figure S4).^31^

The band gap of the TiO_2_-ZnWO_4_ photocatalyst was calculated from the absorbance spectrum of the nanofibers at room temperature with the Tauc plot (Supporting Information, Figure S5). It was found that the *E*_g_ value of the TiO_2_-ZnWO_4_ photocatalyst was 2.76 eV. According to Dette^32^ et al. and Zhang^33^ et al., the valence and conduction bands of TiO_2_ and ZnWO_4_ could be approximately calculated to investigate the charge transfer mechanism by using eqs 1 and 2.^34^
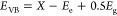
1
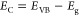
2

It was observed that the conduction band of ZnWO_4_ is lower than that of TiO_2_. Therefore, the excited electron can transfer from the conduction band of TiO_2_ to the conduction band of ZnWO_4_ under UV light irradiation. On the other hand, the holes at the valence band of ZnWO_4_ can also transfer to the valance band of TiO_2_. Both excited electrons and holes are able to react with O_2_ and hydroxide oxide to form super oxide anion and hydroxyl radicals, respectively (Supporting Information, Figure S6).

**Figure 5:**
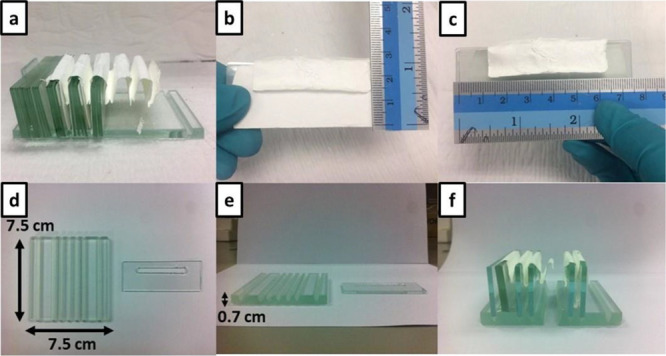
Pictures of pleated TiO_2_-ZnWO_4_ nanofibrous membranes on supporting slides after calcination. Pictures of (a) TiO_2_-ZnWO_4_ nanofibrous membranes after removing supporting slides, (b, c) size of the membrane after calcination, (d, e) a new design stand part and (f) cracked stand part after calcination.

The subsequent configuration of the plate part was to be designed around the optimal nanomembrane thickness and pleating studied above. However, from the previous experiments, it was clear that the nanomembrane shrank to a certain extent upon high-temperature treatment. It was thus important to understand the degree of shrinkage for the subsequent optimal design. Firstly, experiment 5 was repeated, whereas supported glass slides were removed from the plate part (Figure 5a) for nanomembrane size measurement (Figure 5b,c). The average size of each pleated metal oxide membrane was 1.3 cm × 5 cm (original size was about 2.5 cm × 10 cm). In addition, the glass slide dimension was also measured to design the partitioning parameter of the plate part. The function of the plate part was to act as a base and help configure the fixation of the partitioning glass slide support. Therefore, the base was designed to contain rectangular grooves with the width commensurate with that of the designated spacing of the membrane (Figure 5d,e). Up to this point, holes were introduced to the glass slides to improve the ventilation of the model gas (Figure 5d). Unfortunately, with this design, the plate part cracked upon calcination (Figure 5f). It was hypothesized that the presence of the grooves might have introduced in-plane stress concentration and destabilized the physical integrity of the borosilicate base, which thermally expanded to a certain extent during the high-temperature treatment. Then, the grooves’ depth was adjusted so that the base glass was increased from 0.5 to 0.7 cm. This resulted in a stable plate part upon calcination.

**Figure 6:**
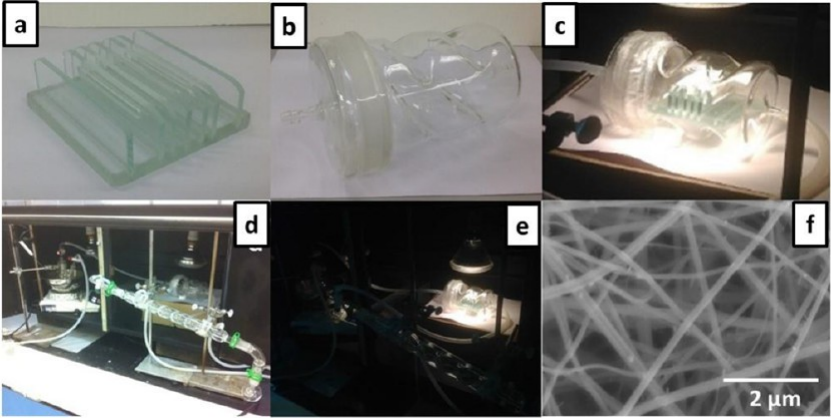
Pictures of the transparent photocatalytic reactor and performing toluene gas degradation experiment. Pictures of (a) the modified plate part, (b) main chamber, (c) the modified plate part inside the main chamber, and experimental set up for toluene decomposition reaction (d) before and (e) after the photocatalytic reaction. (f) SEM image of the TiO_2_-ZnWO_4_ membrane after performing reaction.

The final components were the plate part (Figure 6a) and the main chamber (Figure 6b) that could be assembled into the final structure as shown in Figure 6c, whereas the dimensional congruency between the two components was a critical aspect. The TPR assembly for toluene degradation involved gas inlet and outlet connections as shown on Figure 6d. In this study, gaseous toluene was employed as it represented VOCs from automotive exhaust.^35,36^ During the photocatalytic reaction, visible light was applied throughout the reactor as illustrated in Figure 6e.

### Toluene Gas Degradation under Visible Light Irradiation by TPR with the Photocatalytic TiO_2_-ZnWO_4_ Membrane

For the toluene gas degradation testing procedure, we have modified a laboratory scale experimental set up into a more convenient continuous-flow testing process. Nitrogen gas was employed as a VOC gas carrier,^37^ with a condenser to condense the filtered gas into liquid for subsequent GC–MS analysis. In a typical process, liquid-state toluene was evaporated, passed through the TPR under visible light irradiation, condensed into liquid by the condensation and finally evaluated via GC–MS.^38^ First, the 150 ppm toluene stock solution was prepared and evaporated into the chamber using N_2_ as the gaseous carrier. After the treatment, the gas was condensed into liquid by a cold-water column condenser. The condensate was collected for quantification of toluene concentration by GC/MS. According to reaction kinetics of photocatalytic processes, toluene molecules can be degraded by photogenerated holes and hydroxyl radicals in direct and indirect pathways, respectively.^39^ The results showed 43.91% efficiency of model gas degradation in comparison with the stock solution concentration (Supporting Information, Figure S7). On the other hand, the dark reaction (no light irradiation) was also performed as a reference. It was found that toluene elimination rate was 4.66% which was dramatically lower than the light-assisted reaction. The nonzero value of the dark reaction’s efficiency could be due to surface absorption of the model gas.^37,40^ Even though the toluene degradation performance with the TiO_2_-ZnWO_4_ membrane is not as good as the recently reported chitosan/activated carbon/TiO_2_ catalyst on the PET filter, only visible light irradiation is required for activating the TiO_2_-ZnWO_4_ membrane.^41^ In terms of durability of the TiO_2_-ZnWO_4_ membrane, the all-inorganic membrane after the photocatalytic reaction was characterized by SEM which revealed no physical transformation nor damages as shown in Figure 5f. In addition, the physical characteristics of the TiO_2_-ZnWO_4_ membrane remained intact after performing the photocatalytic reaction for two additional times (Supporting Information, Figure S8). It was noted that the efficiency of model gas degradation could be proportional to the degree of gas-catalyst-light interactions. While increasing the number of membrane’s pleats, gas circulation time and the light intensity could all show positive impact on the reactor’s performance, the above results have successfully demonstrated a proof-of-concept for an unprecedented light-weight and transparent reactor for flow-through solar light conversion. Even though the borosilicate reactor is not as durable as existing photoreactor systems such as corning photoreactor, Firefly system, and Uniqsis PhotoSyn, the borosilicate reactor inherits exceptionally low production cost.^42^ In contrast, the Firefly photochemical reactor uses high-cost quartz reactor tube with cooling water in quartz tubes to operate the photoreaction.

According to the toluene degradation mechanisms, CO_2_ is one of the expected byproducts from the photocatalytic reaction, which can also be harmful to environment.^43^ Therefore, the future design of photocatalytic membranes should include byproduct elimination, capture, or separation.

### Fabrication of the Fiberglass-Supported TiO_2_-ZnWO_4_ Nanofibrous Membrane for the Fixed-Bed Reactor

**Figure 7:**
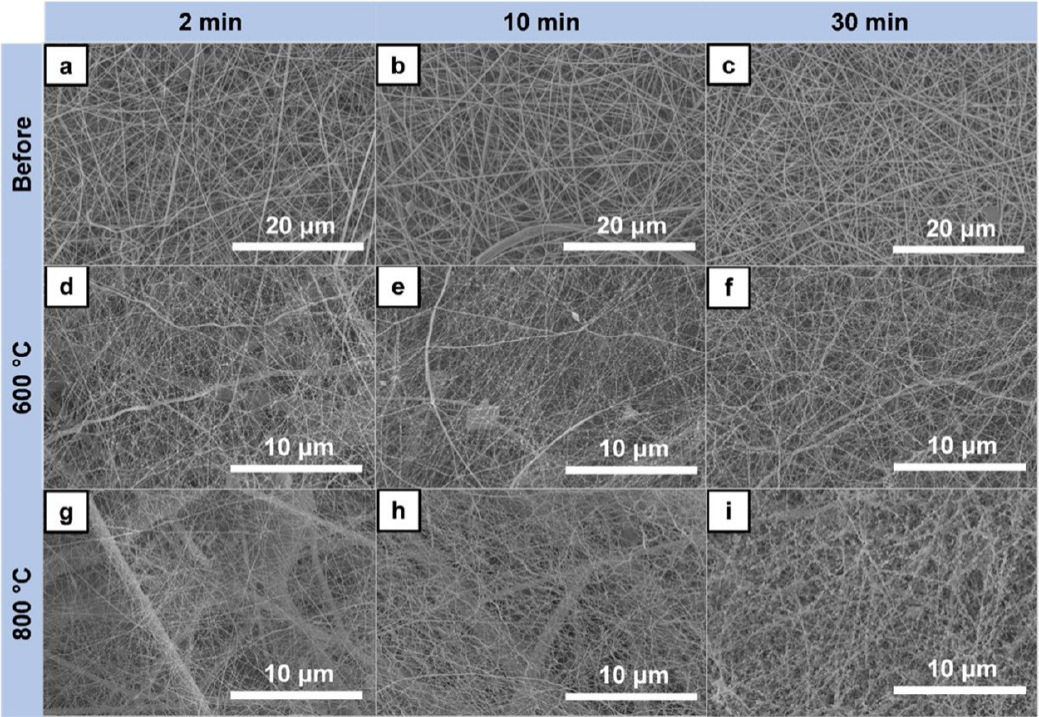
Morphological characterization of electrospun nanofibers on fiberglass membrane at different fabrication time before and after calcination. SEM images of nanofibers on fiberglass membrane (NF/FG) with electrospinning time before calcination for (a) 2 min, (b) 10 min, and (c) 30 min. SEM images of NF/FG after calcination at 600 °C for (d) 2 min, (e) 10 min, (f) 30 min, and at 800 °C, after (g) 2 min, (h) 10 min, and (i) 30 min of electrospinning time.

In addition to the utilization of the TiO_2_-ZnWO_4_ nanofibrous membrane as a photocatalytic membrane inside a photocatalytic converter for the air-phase reaction, the fiberglass-supported TiO_2_-ZnWO_4_ nanofibrous membrane for the fixed-bed reactor was also fabricated to observe the catalyst’s characteristics and photocatalytic performance in MB degradation in solution under natural sunlight. After the electrospinning process, the nanofiber layer became denser depending on the electrospinning time (Figure 7a–c). Therefore, it was clearly seen that the electrospinning method could create the desirable thickness of nanofiber layer, uniformity, as well as satisfactory interweaving with a fiberglass substrate. After the calcination process, the straight-like nanofibers became distorted and were demonstrated as fibrous networks along with tiny rods that appeared on the surface of photocatalytic nanofibers and tended to be larger after increasing of calcination temperature from 600 to 800 °C (Figure 7d–i). In addition, stiffness of the fiberglass substrate increased after the thermal process at 800 °C. It was hypothesized that increasing calcination temperature increased the crystallinity or promoted chemical reactions between TiO_2_-ZnWO_4_ nanofibers and fiberglass membrane. Further increasing the calcination temperature to 1000 °C resulted in degradation of both fiberglass and nanofibrous membrane (Supporting Information, Figure S9).

**Figure 8:**
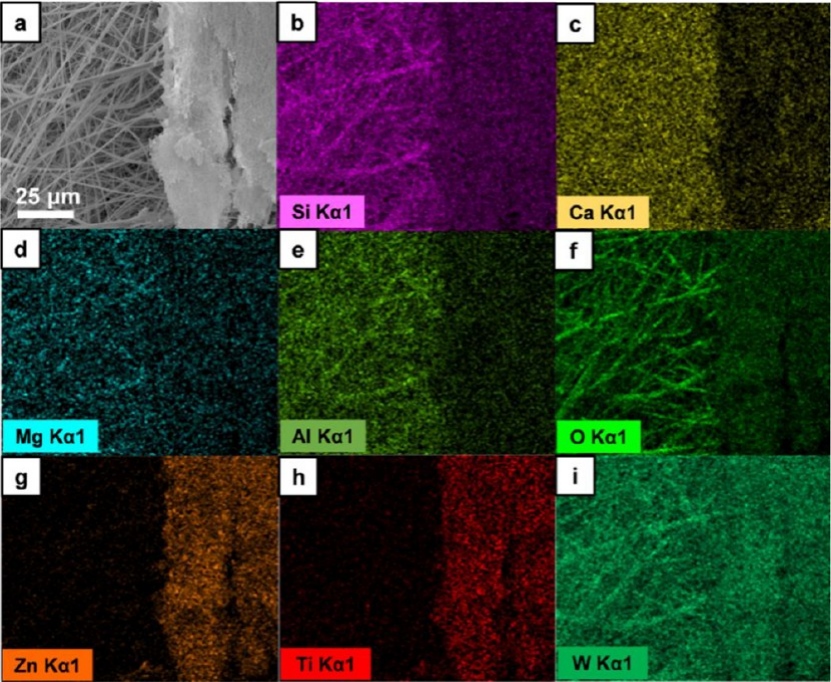
Elemental analysis of fiberglass-supported TiO_2_-ZnWO_4_ membrane after calcination at 800 °C. (a) SEM image of the supported TiO_2_-ZnWO_4_ membrane and elemental mappings of (b) Si, (c) Ca, (d) Mg, (e) Al, (f) O, (g) Zn, (h) Ti, and (i) W.

SEM–EDX characterization was performed to observe the physical and chemical transformation of the fiberglass and fiberglass-supported TiO_2_-ZnWO_4_ nanofibrous membrane (NF/FG). Chemical composition of fiberglass and NF/FG were observed via SEM–EDX elemental mapping. Pristine fiberglass membrane contained Si, Ca, Mg, Al, and O in forms of SiO_2_, CaSiO_3_, MgO, and Al_2_O_3_ (Supporting Information, Figure S10), while fiberglass membrane of calcined NF/FG at 800 °C showed different chemical composition. Figure 8a shows SEM image of fiberglass (left side) and TiO_2_-ZnWO_4_ nanofibrous membrane (right side) of NF/FG at 800 °C. It was revealed that the fiberglass membrane contained W in addition to Si, Ca, Mg, Al, and O (Figure 8b–f). For the TiO_2_-ZnWO_4_ nanofibrous membrane, elemental mapping revealed Zn and Ti as shown in Figure 8g,h. Hence, these investigated results can be significant evidence of atomic exchange or chemical complex formation possibly resulting in well corroboration between photocatalytic nanofiber and fiberglass membrane.

**Figure 9:**
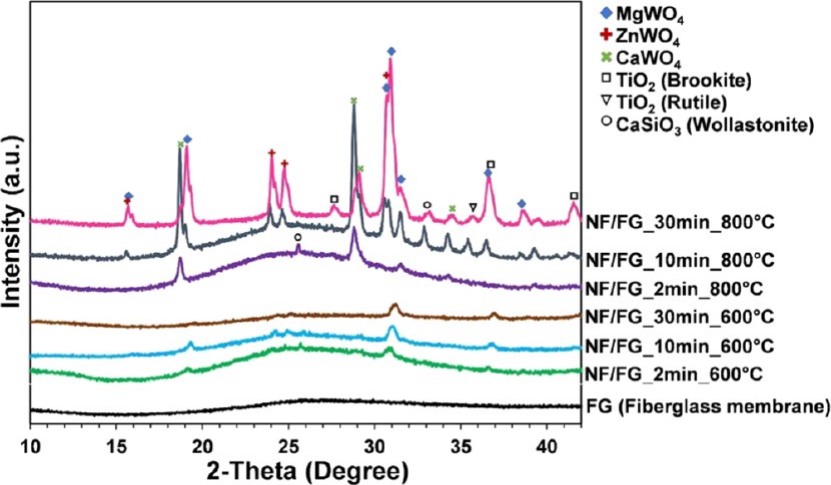
XRD diffraction pattern of fiberglass-supported TiO_2_-ZnWO_4_ (NF/FG) prepared by different electrospinning times and calcination temperatures.

To examine the influence of the calcination temperature, samples with various thermal treatments (without calcination, 600, and 800 °C) and electrospinning time (2, 10, and 30 min) were investigated. In particular, the crystallographic information of prepared NF/FG was investigated by XRD diffraction patterns. As demonstrated in Figure 9, all NF/FG samples prepared without calcination exhibited the amorphous morphology, attributed to component within the as-spun nanofibers and the pristine fiberglass. After calcination at 600 °C, the existence of ZnWO_4_ could be the evident for the formation of TiO_2_-ZnWO_4_. After the thermal treatment at 800 °C, more characteristic peaks were clearly observed for crystalline compounds such as CaWO_4_, MgWO_4_, and CaO (JCPDS card no. 01-071-6152, 00-027-0789, and 00-37-1497). Considering the decomposition temperature of ammonium metatungstate hydrate used as a W source, WO_3_ can be formed during the thermal process at around 380–500 °C.^44^ As a result, the more WO_3_ was introduced into the system, the more CaWO_4_ was obtained through the chemical reaction between WO_3_ from the nanofibers and CaO from the fiberglass. At the same time, the formation of MgWO_4_ could proceed during the thermal treatment in the same way.^45^ The likely formations of CaWO_4_ and MgWO_4_ would be WO_3_ + CaO → CaWO_4_ and WO_3_ + MgO → MgWO_4_, respectively.^46^ On the other hand, the formation of complex structure between Al_2_O_3_ and metal oxides such as WO_3_ was hardly possible due to the high ionic bonding energy of Al_2_O_3_ compared with MgO and CaO. Interestingly, the mixed crystalline peaks of metal oxides, i.e., ZnWO_4_, CaWO_4_, MgWO_4_, CaSiO_3_ (Wollastonite) (JCPDS card no. 00-043-1460), and TiO_2_ (Rutile), were clearly observed after increase of calcination temperature to 800 °C. The characteristic peaks of ZnWO_4_ were presented at around 15.6° (010), 24.8° (110), and 30.8° (111) in the samples prepared by using electrospinning time more than 2 min. The presence of CaSiO_3_, which was found as one of the chemical components of fiberglass membrane analyzed by SEM–EDX, was thermally promoted via increased crystallinity upon the thermal process. In addition, the amount of observed metal-oxide complex was increased as electrospinning time increased, as well agreed with higher peak intensities. The results can once again confirm the success in the synthesis of TiO_2_-ZnWO_4_ composite photocatalyst. It was also noted that the amorphous characteristic patterns of fiberglass membrane depended on the increase of electrospinning time and calcination temperature. The observed results revealed that the interaction between compositions of the fiberglass membrane and as-spun metal oxides precursor during the calcination process resulted in the formation of metal-oxide complex.

**Figure 10:**
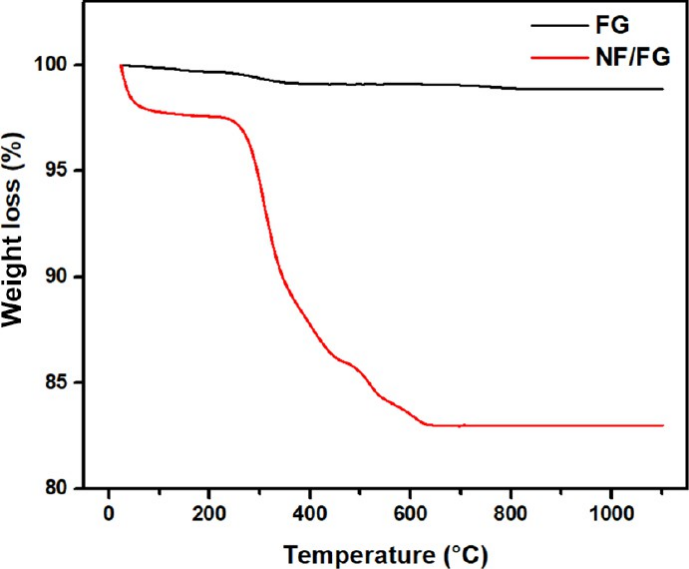
TGA profile of (a) fiberglass membrane and (b) fiberglass-supported TiO_2_-ZnWO_4_ membrane.

Thermogravimetry analysis (TGA) of the fiberglass-supported nanofibrous membrane also confirmed the formation of CaWO_4_ and MgWO_4_. Figure 10 shows TGA of fiberglass membrane and fiberglass-supported nanofibrous membrane. The fiberglass membrane did not show significant weight loss (less than 2%) throughout the temperature range from room temperature to 1100 °C which proved its high thermal stability. On the other hand, TGA of the fiberglass-supported TiO_2_-ZnWO_4_ membrane showed several steps of weight loss. The first weight loss around room temperature was contributed to moisture within the nanofibrous membrane. Subsequently, a major weight loss around 350 °C was concerned with PVP degradation and WO_3_ formation. Two steps of weight losses around 500 and 600 °C could be attributed to ZnWO_4_, CaWO_4_, and MgWO_4_ formations.^47^

Following the successfully fabrication of the supported catalysts, degradation of MB experiment under natural sunlight was performed to demonstrate their photocatalytic properties. Concerning their dye adsorption ability, prepared samples, i.e., NF/FG with electrospinning time of 2, 10, and 30 min calcined at 600 °C and with electrospinning times of 30 min calcined at 800 °C were chosen to be tested, compared with the original fiberglass membrane (Supporting Information, Figure S11). The NF/FG samples were soaked in 100 mL of MB (5 ppm) for 2 h without illumination. Herein, the dye adsorption ability was calculated through the following: %DA = 100 × [(*A*_0_ – *A*)/*A*_0_], where *A*_0_ and *A* are initial absorbance intensity and intensity measured after 2 h of soaking time, respectively. The results suggested that %DA was reduced in NF/FG_600 °C with 2 min of electrospinning times, whereas it tended to increase continuously after extension of electrospinning times to 10 and 30 min, respectively. This implied that at 2 min of electrospinning times low amount of photocatalytic fiber was introduced on the fiber glass surface. As a result, the achieved rigid surface with insufficiency photocatalytic fiber of prepared sample after calcination demonstrated poor dye adsorption ability. For 10 and 30 min of electrospinning times, the existence of thicken photocatalytic fiber could promote the dye adsorption ability. For influence of calcination temperature, with 30 min of electrospinning times, the sample prepared at 800 °C performed lower dye adsorption ability than the sample prepared at 600 °C. It could be noted that the surface of prepared sample can possibly become more rigid upon the increase of calcination temperature resulting in lower dye adsorption ability.

**Figure 11:**
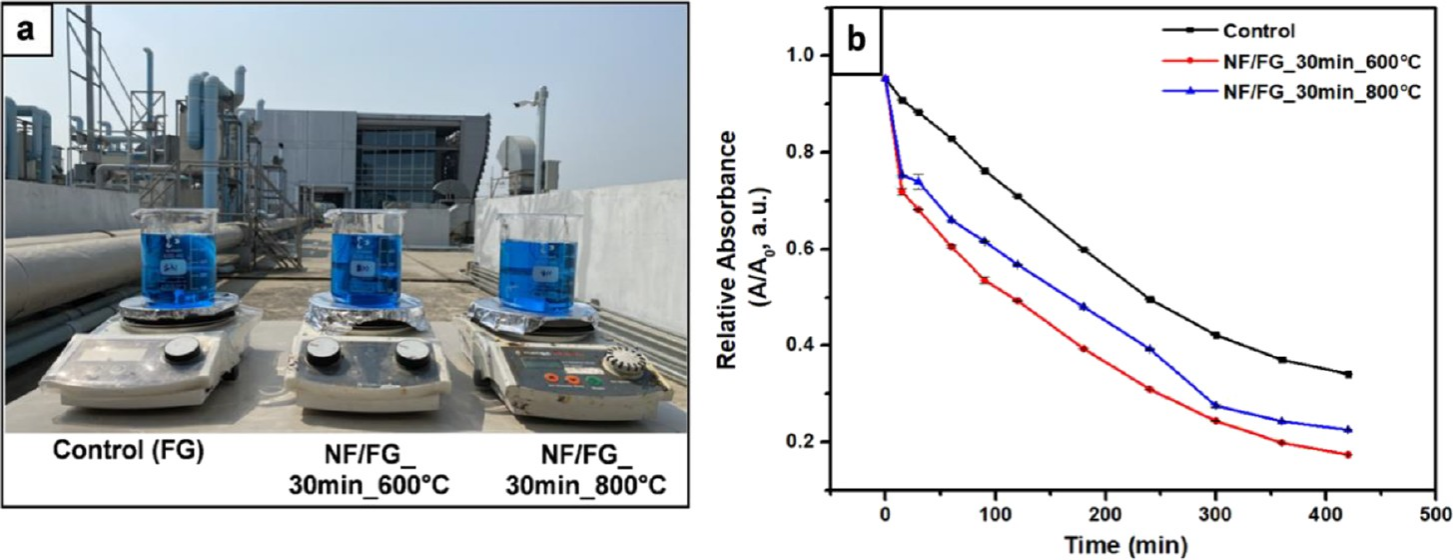
(a) Picture of MB degradation experiment under natural sunlight and (b) photocatalytic activity of control (fiberglass membrane), NF/FG_600 °C, and NF/FG_800 °C.

Figure 11a illustrates the photocatalytic activity test under sunlight irradiation. Herein, MB was adopted as a model of organic pollutant. Photocatalytic reaction kinetics of MB degradation by the TiO_2_-ZnWO_4_ photocatalytic membrane generally concerns the generation of holes and electrons by light activation on the photocatalyst. The holes may react with surface hydroxyls of TiO_2_ or water which generate reactive hydroxyl radicals, while electrons may react with electron acceptors on the photocatalyst surfaces or oxygen which result in super oxide anion. Both hydroxyl radicals and super oxide anion can then degrade MB that absorbed on the photocatalyst surface into CO_2_ and H_2_O.^31,48^ The couple plate of prepared catalyst-coated fiberglass membrane was put in the 500 mL of MB. In addition, the couple plate fiberglass without any nanofibrous catalyst was studied as a control sample (MB degradation under natural sunlight is shown in Figure S12a, Supporting Information). Interestingly, the results suggested that a control sample presented MB dye adsorption ability which can be an advantage for the overall dye removal process. At the end of the reaction time (average light intensity throughout the reaction was 79,600 Lux), it could be clearly seen that NF/FG samples were performed around two times better than control filter membrane in degrading MB under sunlight irradiation (Figure 11b). Although calcination at 800 °C resulted in higher crystallinity of photocatalysts, the size of catalytic fiber tended to be larger and surface area became lower. Besides, the cracks and rigid surface of catalytic fiber were achieved at this calcination temperature, as well as for fiberglass membrane. The results were well agreed with lower dye adsorption ability compared with the sample calcined at 600 °C (with the same electrospinning time of 30 min). As a result, the photocatalytic activity of NF/FG_800 °C was lower than that of NF/FG_600 °C. Morphological structures of the NF/FG_600 °C remained intact after performing the MB degradation reaction (Supporting Information, Figure S12b). Even though the photocatalytic performance of the NF/FG membrane may be inferior to its powder form and recently reported photocatalysts for MB degradation such as ZnO/N-CQD,^49^ Zn:SnO_2_/CCAC,^50^ and graphene based NiMnO_3_/NiMn_2_O_4_,^51^ the NF/FG membrane presents distinct advantages over mentioned photocatalytic systems in terms of facile photocatalyst recovery and utilization in actual environment.

## Conclusions

Pleated TiO_2_-ZnWO_4_ and fiberglass-supported TiO_2_-ZnWO_4_ nanofibrous membranes were successfully fabricated via electrospinning for air and water pollutant decomposition, respectively. For toluene gas decomposition, the borosilicate TPR was invented to support the pleated TiO_2_-ZnWO_4_ membrane. Under visible light irradiation, the TPR was able to decompose toluene employed as a model gas with more than 40% efficiency without changing the physical characteristics of the membrane. On the other hand, fiberglass-supported TiO_2_-ZnWO_4_ nanofibrous membrane could be applied in fixed-base reactor for MB degradation under natural sunlight. The result showed that the fiberglass-supported TiO_2_-ZnWO_4_ could decompose 5 ppm MB solution more than 70% within 3 h. Therefore, it could be worth noting that the pleated TiO_2_-ZnWO_4_ and fiberglass-supported TiO_2_-ZnWO_4_ nanofibrous membrane achieved in this research could be one of the promising artificial photocatalyst for environmental applications. However, several experiments may be conducted and evaluated before utilizing the photoreactor and the fiberglass-supported TiO_2_-ZnWO_4_ membrane for decomposing pollutants in a pilot scale. In particular, gas flow speed and pollutant concentration should be optimized with the photoreactor system, while varieties of resulting intermediate may be evaluated for the fiberglass-supported TiO_2_-ZnWO_4_ membrane.

## Materials and Methods

### Materials

Polyvinylpyrrolidone (PVP, *M*_w_ ∼ 1,300,000, Fluka), ammonium metatungstate hydrate (AMT, (NH_4_)_6_-H_2_W_12_O_40_·*x*H_2_O, Sigma-Aldrich), zinc acetate dihydrate (ZAH, C_4_H_6_O_4_Zn·2H_2_O, ≥98.0%, Sigma-Aldrich), titanium(IV) isopropoxide solution (TIP, C_12_H_28_O_4_Ti, ≥97.0%, Sigma-Aldrich), toluene (Sigma-Aldrich), ethanol (99.8%, Sigma-Aldrich), *N*,*N*-dimethylformamide (DMF ≥99.0%, Sigma-Aldrich) and glacial acetic acid (Sigma-Aldrich), were of analytical grade and used as received. Glass microfiber (Grade 934-AH, diameter 70 mm, Sigma-Aldrich) and glass slides (25.4 × 76.2 mm, 1–1.2 mm thickness, Sigma-Aldrich) were used as received.

### Sol–Gel Electrospinning Solution Preparation

#### Preparation of Ammonium Metatungstate Hydrate and Zinc Acetate Dihydrate Electrospinning Solution

The solution was prepared by dissolving PVP (3 g) in ethanol (30 mL) under magnetic stirring for 30 min. In two separate beakers, AMT (0.6 g) was added into DMF (6 mL) under magnetic stirring for 10 min while ZAH solution was prepared by dissolving ZAH (0.6 g) in DMF (6 mL) under magnetic stirring for 10 min. Finally, all three solutions were mixed under magnetic stirring for 10 min prior to electrospinning.

#### Preparation of Ammonium Metatungstate Hydrate, Zinc Acetate Dihydrate, and Titanium(IV) Isopropoxide Electrospinning Solution

The electrospinning solution was freshly prepared under fume hood.^28^ Specifically, PVP (30 g) was dissolved in ethanol (300 mL) under magnetic stirring for 20 min. Two metal salt solutions were prepared separately. AMT (6 g) was dissolved in DMF (60 mL), while ZAH (6 g) was dissolved in DMF (60 mL) to prepared 10% w/v AMT and ZAH solutions, respectively. The AMT solution was then added into the PVP solution before slowly adding the ZAH solution. After that, TIP solution (60 mL) was added into the solution, followed by concentrated acetic acid (60 mL).

#### TiO_2_-ZnWO_4_ Nanofibrous Membrane Fabrication for Flow-Through a Transparent Photocatalytic Converter

**Scheme 2:**
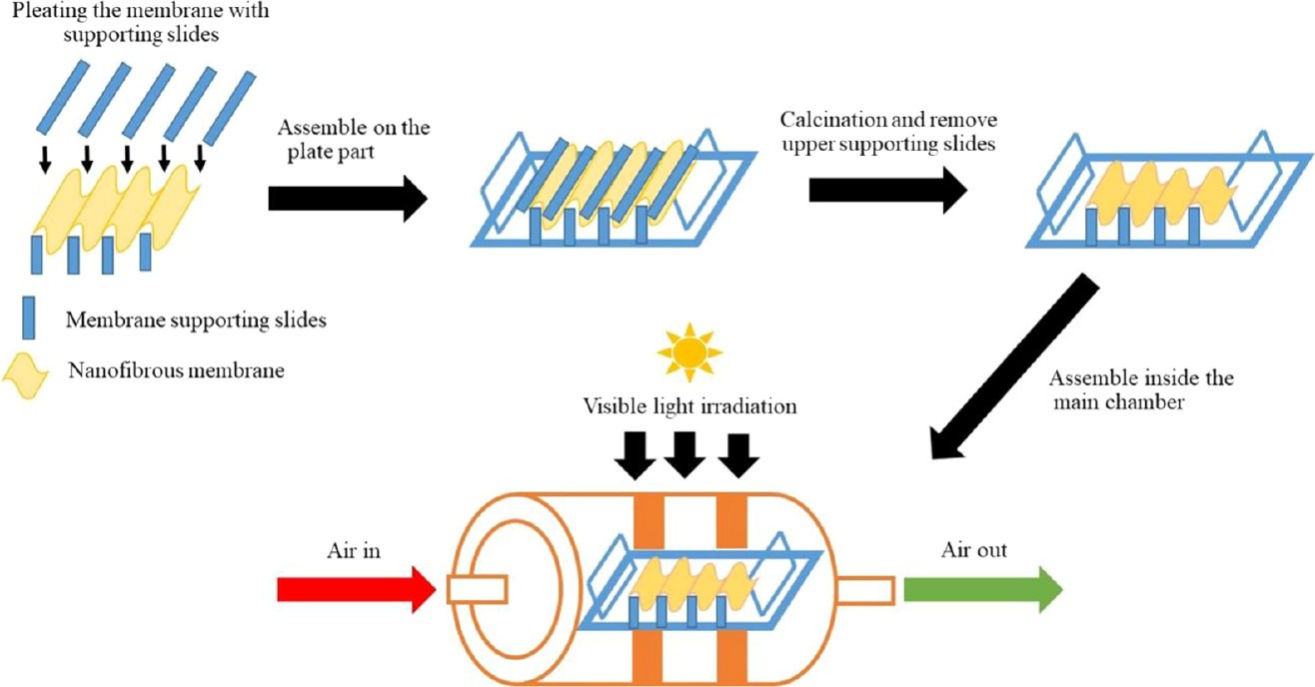
Composition and Assembly Processes of the Transparent Photocatalytic Reactor (TPR)

Fabrication of TiO_2_-ZnWO_4_ nanofibrous membrane in a transparent photocatalytic converter (TPR) was divided into nanofibrous membrane fabrication and transparent photocatalytic converter construction. In a first part, the nanofibrous membrane was fabricated by a pre-pilot scale Nanospider machine (NS LAB 500, Elmarco, Czech Republic). Firstly, the electrospinning solution (350 mL) was added into a large cylindrical chamber inside the Nanospider machine. Prior to performing the electrospinning process, the distance between electrode and solution, voltage, and rotating electrode were adjusted at 18 cm, 50 kV, and 8 rpm, respectively. In a second part, the TPR composed of a plate part, membrane supporting slides, and a main chamber which made of borosilicate glasses and tubes. First, the plate part was designed as a rectangular flat shape of 0.7 cm in thickness and 7.5 cm long and composed of 7 cavities for supporting slide assembly. Each cavity was 0.3 mm in width, 0.5 mm in depth, and 0.3 m in thickness or spacing. Second, the membrane supporting slide was designed as a rectangle flat shape with a hole for supporting a pleated nanofibrous membrane and air flow during the flow-through reaction. The dimension of the slide was 7.5 cm in length and 2.1 cm in width. Finally, the main chamber (80 mm inner diameter, 85 mm outer diameter and 11 cm long) was designed to contain the plate part and facilitate air ventilation throughout the assembly. To assemble the nanofibrous membrane into the TPR, the membrane was firstly pleated on the plate part with the membrane supporting slides. The pleated membrane on the plate part was then calcined inside a furnace (Nabertherm GmbH, model: LT 15/12/P320) at 600 °C for 4 h. After the calcination process, the plate part was inserted inside the main chamber (Scheme 2).

### Fiberglass0Supported TiO_2_-ZnWO_4_ Nanofibrous Membrane Fabrication for Fixed-Bed Reactor

The fiberglass-supported nanofibrous membrane was fabricated via Nanospider machine using fiberglass as a support collector. First, the electrospinning solution (350 mL) was added into a large cylindrical chamber inside the Nanospider machine. To fabricate the nanofibrous membrane onto the fiberglass, the fiberglass membranes were attached on the collector prior to the electrospinning process. During the electrospinning process, the distance between the electrode and solution, voltage, and rotating electrode was adjusted at 18 cm, 50 kV, and 8 rpm, respectively. The electrospinning time was varied at 2, 10, and 30 min. After the electrospinning process, the fiberglass-supported nanofibrous membrane was sandwiched before calcination in a furnace at 600, 800 and 1000 °C for 4 h.

### Toluene Gas Degradation under Visible Light Irradiation

**Scheme 3:**
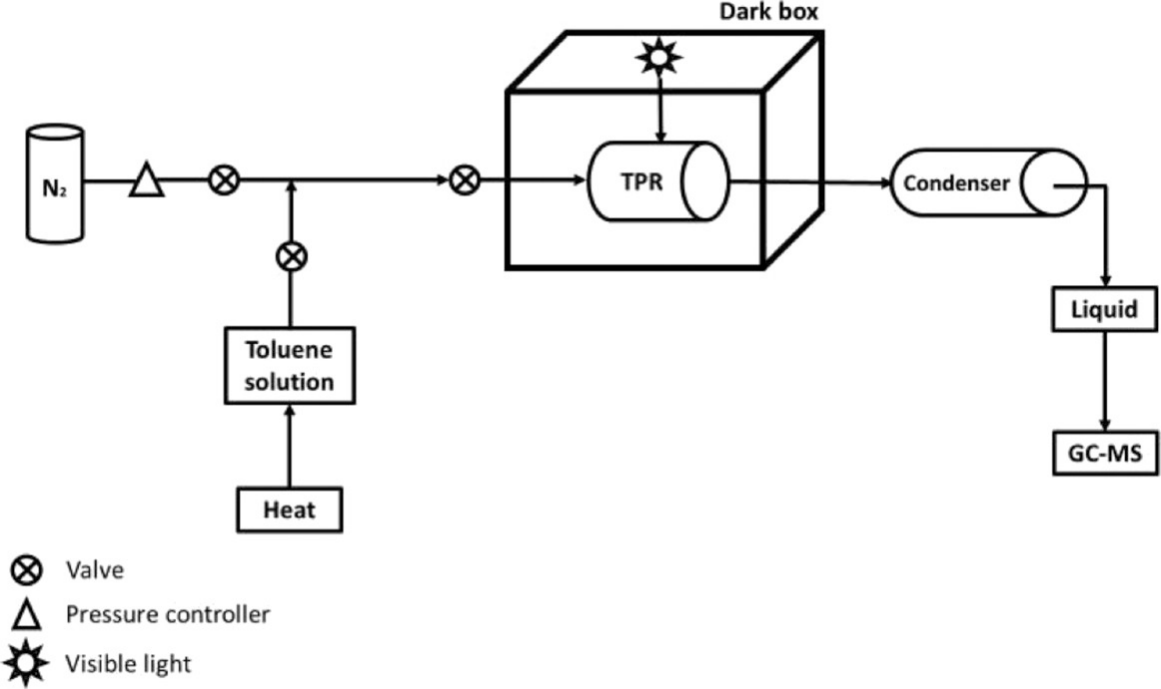
Continuous-Flow Experimental Set Up for Toluene Gas Degradation

The photocatalytic experiment was performed under visible light irradiation in a fume hood (Scheme 3). Experimentally, a three-neck flask was connected with the TPR and a condenser via silicon tubes. The TPR was placed under a visible light bulb with a fixed distance at 10 cm. Nitrogen gas (N_2_) was then directed through the chamber for 20 min at a constant flow rate of 0.2 mbar for equilibration. After closing all tube valves, a 150 ppm toluene solution was prepared by diluting 150 μm toluene in 100 mL 1-propanol. Subsequently, the toluene solution was poured into the three-neck flask, followed by heating at 110 °C. The inlet valves were then opened to allow the continuous flow of toluene carried by N_2_ gas at 0.2 mbar throughout the chamber in once through mode. Two visible light bulbs (120 W) were turned on immediately after the start of gas flow. The photocatalytic reaction was continued for 3 h before collecting the toluene solution from the condenser. Finally, the remaining of the model toxin was then quantified by GC–MS.

### MB Degradation under Natural Sunlight

The photocatalytic reaction was performed under natural sunlight with average sunlight intensity of 590,000 Lux (Heavy duty light meter, Extech instrument, model 407026) throughout the experiment. Firstly, a selected fiberglass-supported TiO_2_-ZnWO_4_ membrane was fixed in the middle of a 600 mL beaker by sieve. Subsequently, 5 ppm MB solution (500 mL) was added into the beaker before placing on the magnetic stirrer under natural sunlight. During the reaction, 2 mL aliquot of the methylene solution was collected every 1 h for measure a concentration via UV–vis spectrophotometer at 663 nm.

### Physical and Chemical Characterizations

Physical and chemical characteristics of nanofibrous membrane were characterized by scanning electron microscopy (SEM, SU5000, 10 kV, working distance at 5 mm; SEM–EDX, 20 kV, working distance at 10 mm) and high-resolution transmission electron microscopy (HR-TEM, JEOL JEM-2010). Band gap (*E*_g_) of TiO_2_-ZnWO_4_ photocatalyst was measured by UV–vis spectrophotometer (Perkin Elmer, model: Lambda 650) and calculated with the Tauc plot.^52−54^ XRD patterns were collected in a range from 10 to 80° 2θ with a Cu source (λ = 1.54 Å; 40 kV; 40 mA) by X-ray diffractometer (Bruker, D8 Advance). Thermogravimetric analysis (TGA) was conducted under air at a heating rate of 2 °C/min from room temperature to 1100 °C (Shimadzu/DTG-60AH). Toluene concentration was measured using gas chromatography/mass spectroscopy (GC/MS: QP2010 Ultra, library: NIST14, column: DB-5, Shimadzu). MB concentration was measured via UV–vis spectrophotometer (Perkin Elmer, model: Lambda 650).

## Data Availability

All data are available upon publication.

## Supporting Information Available

SEM images of the ZnWO_4_ nanofibrous membrane; pictures of the trial pleating experiments; XRD pattern of TiO_2_-ZnWO_4_ nanofibrous membrane; calculated band gap of TiO_2_-ZnWO_4_ photocatalyst; schematic diagram of charge transfer mechanism of TiO_2_-ZnWO_4_ photocatalyst under light irradiation; GC–MS spectrums of toluene concentration; SEM images of TiO_2_-ZnWO_4_ membrane after performing second and third photocatalytic reactions; picture and SEM image of fiberglass-supported nanofibrous membrane; FE-SEM images of fiberglass membrane (FG) with elemental mappings and EDX spectrum; relative absorbance intensities (*A*/*A*_0_) of MB dye in the presence of prepared samples; relative absorbance intensities (*A*/*A*_0_) of MB dye degradation under natural sunlight (PDF)
