# Supplementary material for: Supported TiO2-ZnWO4 Photocatalytic Nanofibrous Membranes for Flow-Through and Fixed-Bed Reactors
Source: ACS Omega. 2023 Aug 9;8(33):30389–401. doi: 10.1021/acsomega.3c03527 (PMC10448639; doi:10.1021/acsomega.3c03527)
Supplement: Supplementary file 1 — ao3c03527_si_001.pdf [file ao3c03527_si_001.pdf]

# Electronic Supplementary Information

## Supported $TiO_2$-$ZnWO_4$ Photocatalytic Nanofibrous Membranes for Flow-Through and Fixed-bed Reactors

Nakarin Subjalearndee, Pasinee Panith, Tanaporn Narkbuakaew, Pech Thongkam, and Varol Intasanta*

National Nanotechnology Center, National Science and Technology Development Agency, 111 Phahonyothin Road, Klong Nueng, Klong Luang, Pathumthani, 12120, Thailand.

* Corresponding author: varol@nanotec.or.th

## Supporting information

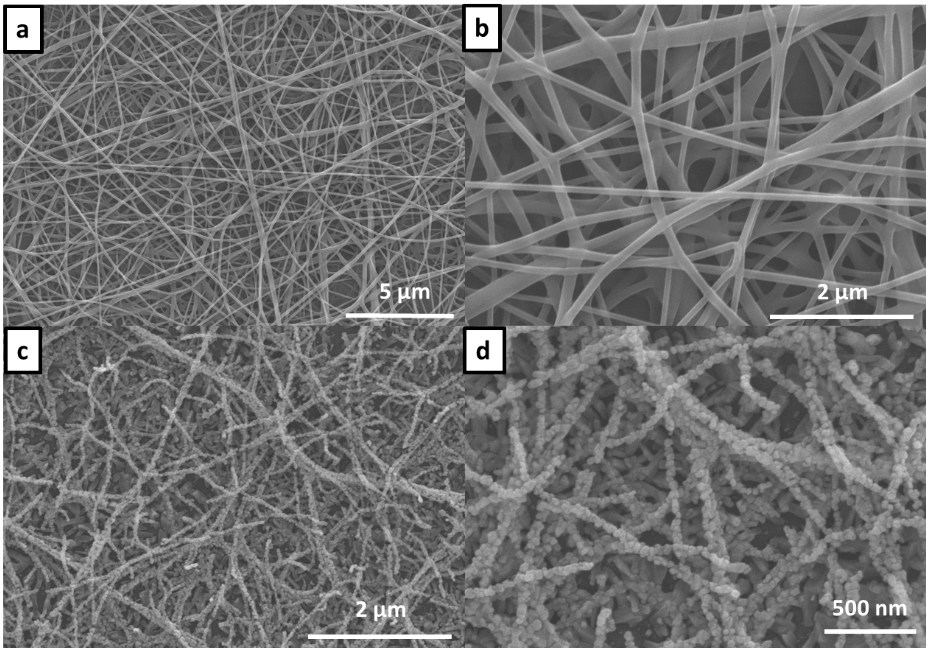


**Figure S1.** SEM images of nanofibers fabricated from AMT and ZAH precursors (a and b) before and (c and d) after calcination at 600 °C.

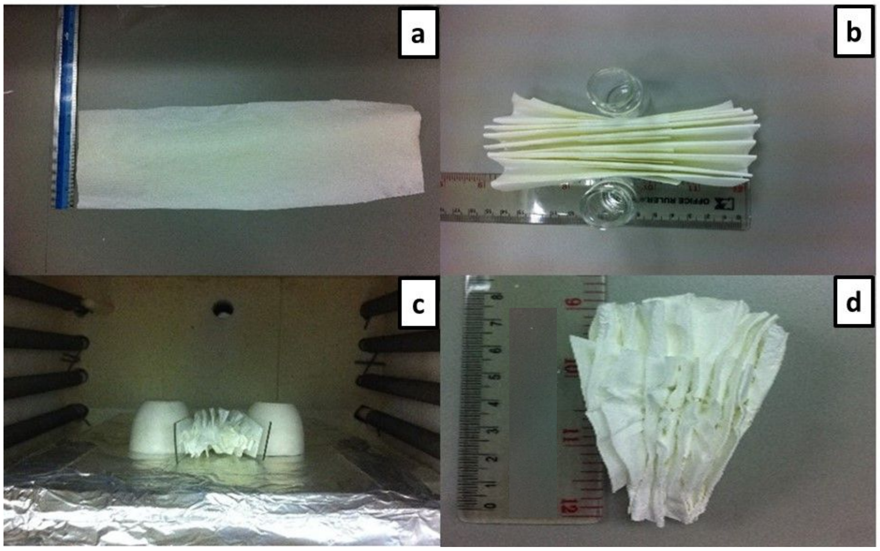


**Figure S2.** Pictures of (a) as-spun nanofibrous membrane, (b) the membrane after pleating by using fiber glass as supports, the membrane after calcination (c) inside and (d) outside a furnace.

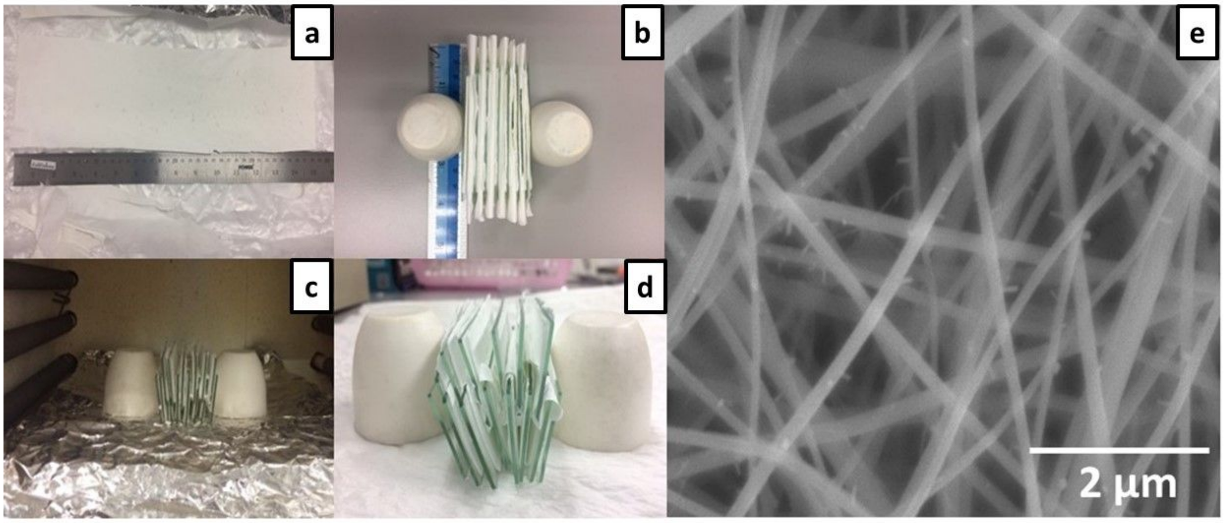


**Figure S3.** Pictures of (a) as-spun nanofibrous membrane, (b) the membrane after pleating by using fiber glasses and glass slides as supporting slides, the membrane after calcination (c) inside and (d) outside a furnace and (e) SEM image of the membrane after calcination.

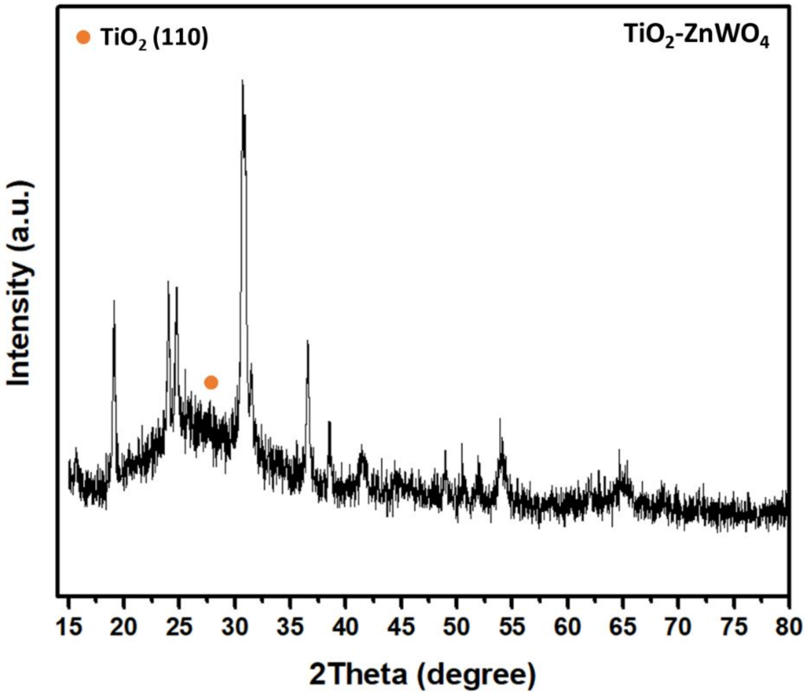


**Figure S4.** XRD pattern of $TiO_2$-$ZnWO_4$ nanofibrous membrane

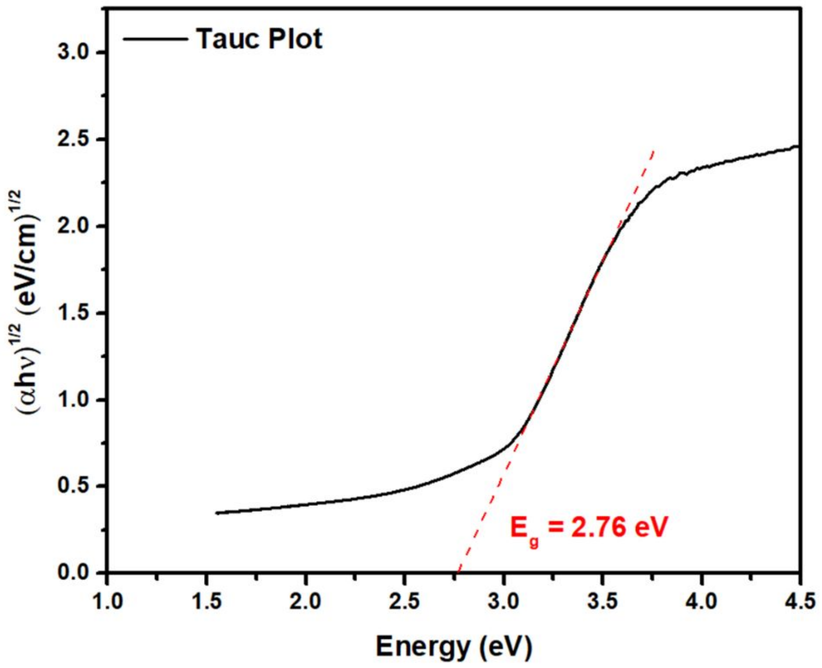


**Figure S5.** Calculated band gap of $TiO_2$-$ZnWO_4$ photocatalyst.

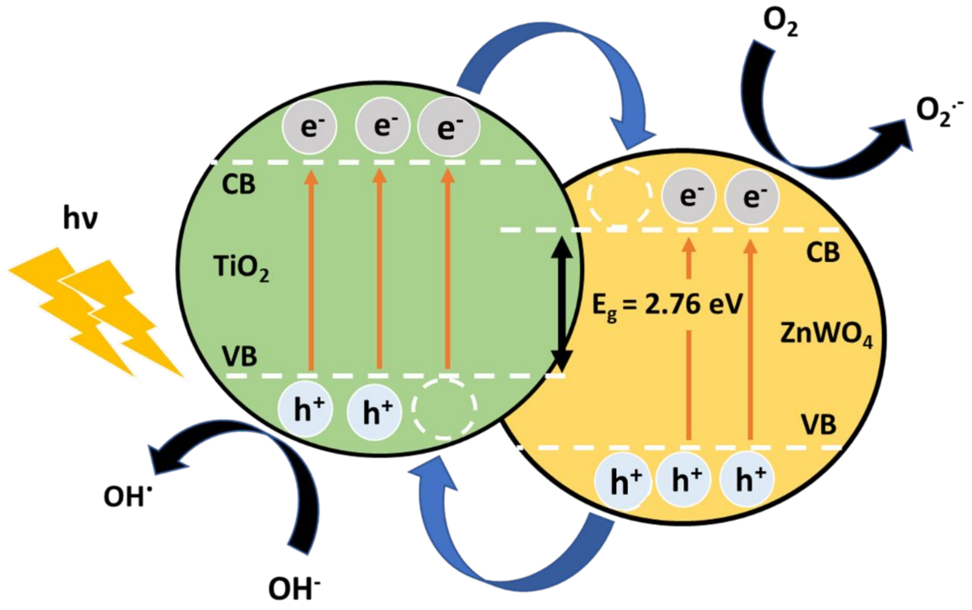


**Figure S6.** Schematic diagram of charge transfer mechanism of $TiO_2$-$ZnWO_4$ photocatalyst under light irradiation.

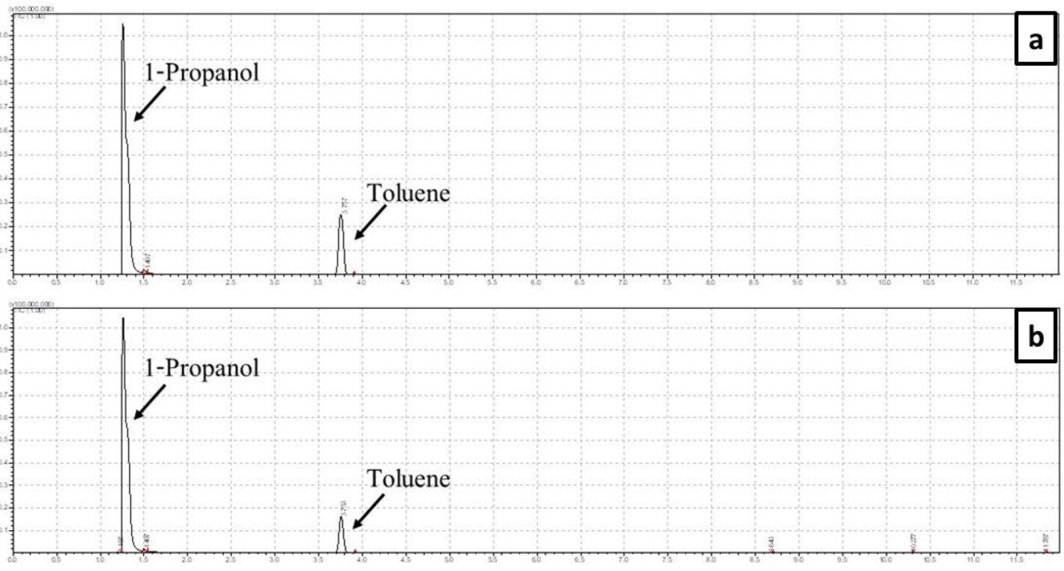


**Figure S7.** GC-MS spectrums of toluene concentration (a) before and (b) after photocatalytic reaction under visible light irradiation.

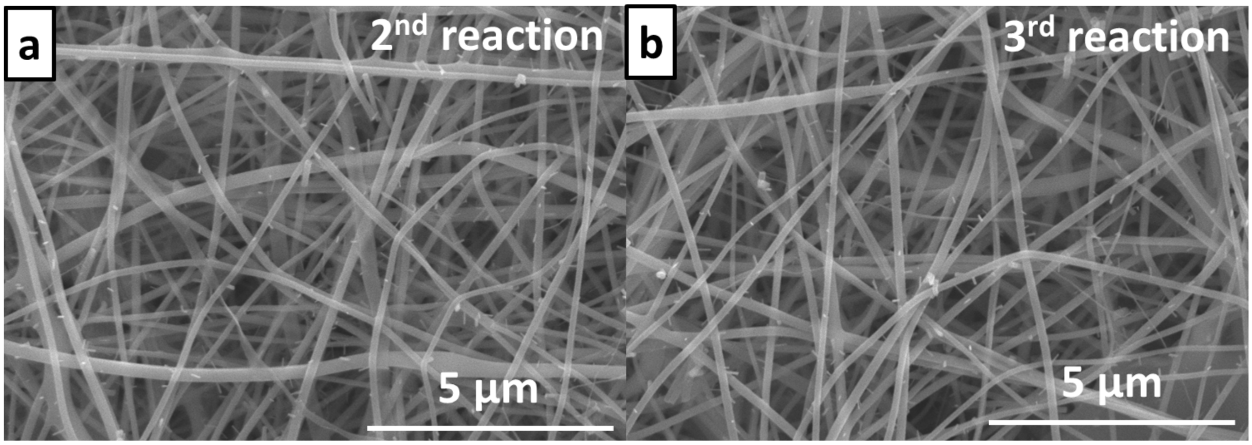


**Figure S8.** SEM images of $TiO_2$-$ZnWO_4$ membrane after performing (a) $2^{nd}$ and (b) $3^{rd}$ photocatalytic reactions.

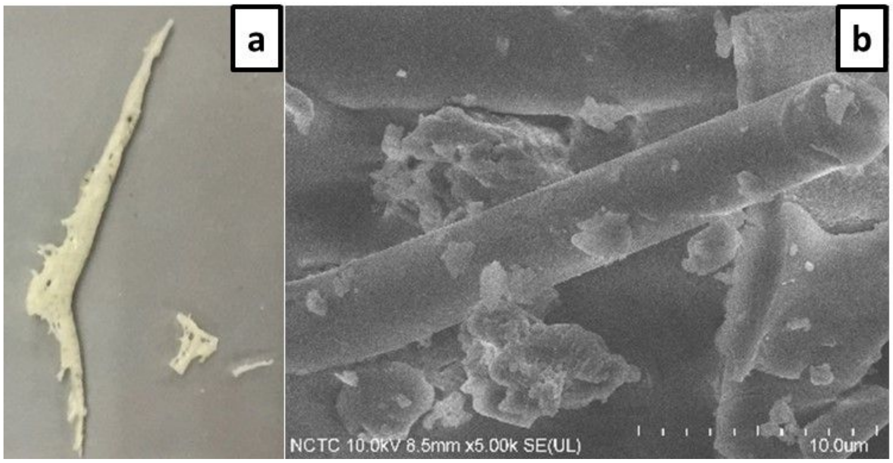


**Figure S9.** (a) Picture and (b) SEM image of fiberglass supported nanofibrous membrane after calcination at 1,000 °C.

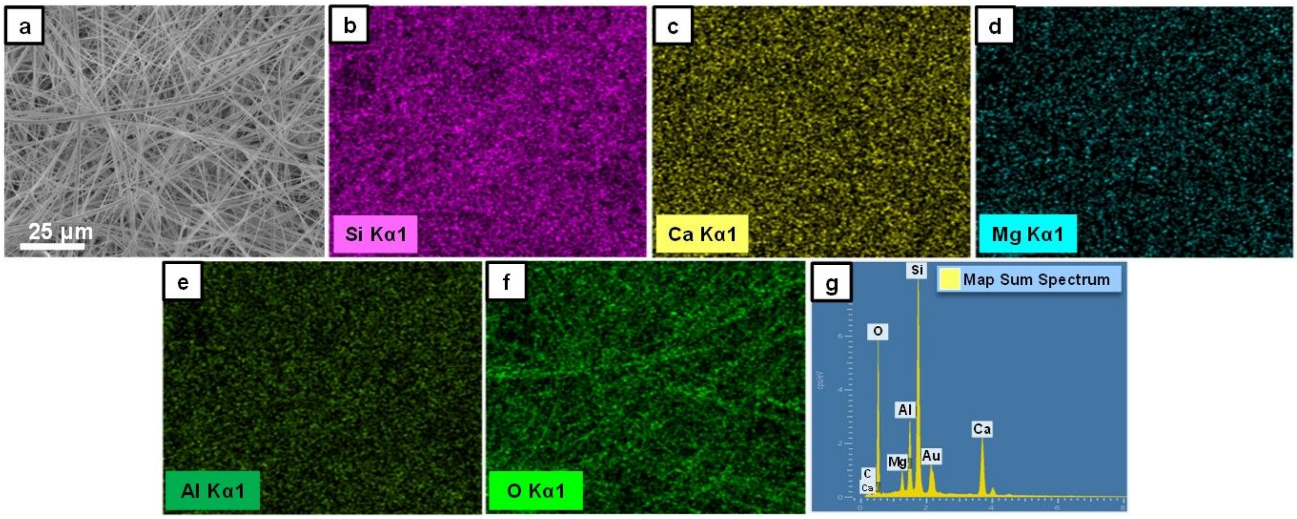


**Figure S10.** FE-SEM images of (a) fiberglass membrane (FG) with elemental mappings (b-f) of Si, Ca, Mg, Al, and O, and (g) EDX spectrum.

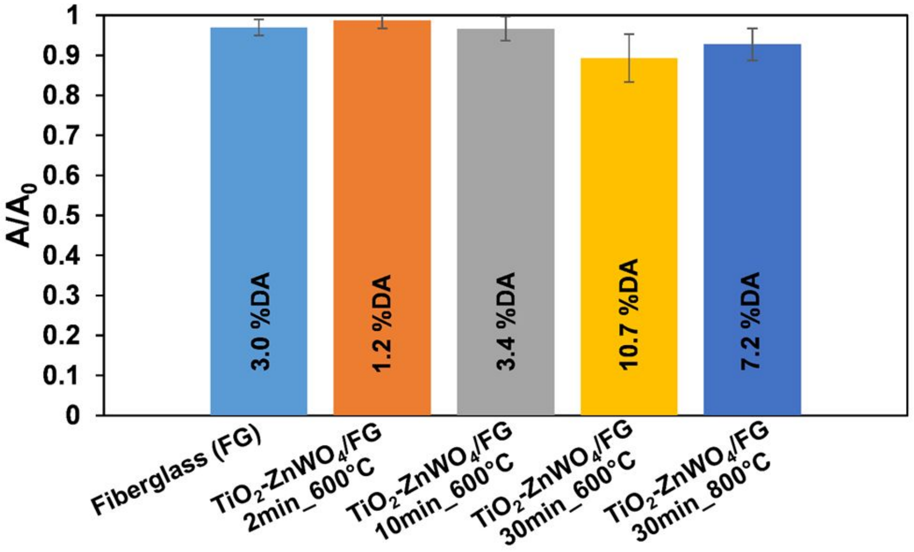


**Figure S11.** Relative absorbance intensities ($A/A_0$) of MB dye in the presence of prepared samples for 2 h without illumination.

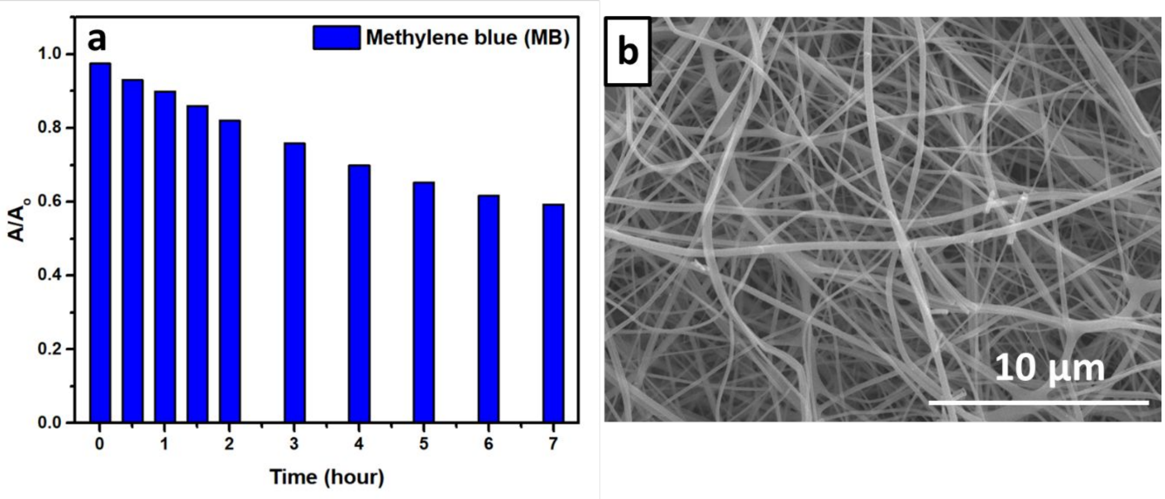


**Figure S12.** (a) Relative absorbance intensities ($A/A_0$) of MB dye degradation under natural sunlight for 7 hours (average light intensity throughout the reaction was 62,400 Lux) and (b) morphological structures of NF/FG_600 °C membrane after performing the MB dye degradation under sunlight irradiation.
